# The trade-off between health system resiliency and efficiency: evidence from COVID-19 in European regions

**DOI:** 10.1007/s10198-023-01567-w

**Published:** 2023-02-02

**Authors:** Alvaro Almeida

**Affiliations:** 1 Cef.up Center for Economics and Finance at UPorto, Rua Dr. Roberto Frias, 4200-464 Porto, Portugal; 2 Faculdade de Economia, Universidade do Porto, 4200-464 Porto, Portugal

**Keywords:** Health systems, Resilience, Efficiency, COVID-19, I10, I18

## Abstract

The objective of this paper was to investigate the existence of a trade-off between health system resilience and the economic efficiency of the health system, using data for 173 regions in the European Union and the European Free Trade Association countries. Data Envelopment Analysis was used to measure the efficiency of regional health systems before the COVID-19 pandemic. Then, a spatial econometrics model was used to estimate whether this measure of efficiency, adjusted for several covariates, has a significant impact on regional health system resilience during the COVID-19 pandemic, measured by the number of COVID-19 deaths per hundred thousand inhabitants. The results show that COVID-19 death rates were significantly higher in regions with higher population density, higher share of employment in industry, and higher share of women in the population. Results also show that regions with higher values of the health system efficiency index in 2017 had significantly higher rates of COVID-19 deaths in 2020 and 2021, suggesting the existence of a trade-off between health system efficiency and health system resilience during the COVID-19 pandemic.

## Introduction

Economic efficiency of the health system has been one of the central preoccupations of health policymakers and economic analysts for most of the second decade of the twenty-first century, as the global financial and economic crisis of the end of the previous decade had exposed health systems to financial pressures and concerns over long-term economic and financial sustainability. For instance, the importance of reforms to improve economic efficiency of health systems featured prominently on the OECD’s Health at a Glance 2013’s Editorial [1] and 2019’s Executive Summary [2]. Improving health system efficiency may be simply presented as improving the outcomes for a given set of inputs, or to reduce the inputs necessary to achieve a given level of outcomes. Increased efficiency allows to maintain health gains with a moderate use of resources, a key policy goal for budget constrained policymakers.

The outbreak of the COVID-19 pandemic changed the focus from health system efficiency to health system resilience. The 2021 edition of the OECD’s Health at a Glance [3] was presented under the headline “COVID-19 pandemic underlines need to strengthen resilience of health systems”. Health system resilience is a relatively recent concept, and descriptions of health system resilience vary in the existing literature [4], but it is usually defined as the capacity to prepare for and effectively respond to crises whilst retaining core health system functions when a crisis hits [5].

In complex adaptive systems, such as the health system, tensions exist between efficiency and resilience [6]. A trade-off between system efficiency and system resilience has been documented in ecological, business, and other literature (for example, [7, 8] or [9]). In health systems, a focus on efficiency may hinder the resilience of the system through at least three channels. First, resiliency requires *surge capacity*, the policies, practices, and systems necessary to accommodate a surge of patients during a public health emergency [10]; building surge capacity is likely to require keeping excess resources that will not be fully employed in non-crisis periods, thus reducing efficiency. Second, building resilience implies investment in crisis preparation (for example, reinforcing infrastructure or information systems, developing and training contingency plans); when the focus is on efficiency, these investments may be seen as unnecessary or unjustifiably costly [11]. Third, efficiency usually implies streamlining and specialization, while resilience requires flexibility and redundancy [12].

The possible existence of a trade-off between health system resiliency and economic efficiency is a critical question for policymakers. If such a trade-off exists, then policymakers face a difficult choice between preparing the health system to better respond to the next crisis or increasing health outcomes in the present. If the trade-off is not relevant, then reforms to improve economic efficiency of health systems may continue without endangering the system’s capacity to respond to the next crisis.

The objective of this paper is to investigate the existence of a trade-off between health system resilience and economic efficiency in European health systems. Using data for 173 regions in the European Union and the European Free Trade Association countries, Data Envelopment Analysis is used to measure the efficiency of regional health systems before the COVID-19 pandemic. Then, a spatial econometrics model is used to estimate whether this measure of efficiency, adjusted for several covariates, had a significant impact on regional health system resilience during the COVID-19 pandemic, measured by the number of COVID-19 deaths per hundred thousand inhabitants. The COVID-19 death rate is used as the indicator of health system resiliency because avoiding death was the key health policy goal during the COVID-19 pandemic. Thus, health systems with lower COVID-19 death rates were the systems that more effectively responded to the COVID-19 health crisis, i.e., the health systems with lower death rates were the more resilient health systems (excess mortality would likely be a better indicator of health system resilience than COVID-19 death rates—see Box 1.2 in [13] for a discussion—but comparable data at a regional level were not available).

The level of analysis in this study is regional and not national because evidence showed that the impact of the COVID-19 crisis differed sharply not only across countries but also across regions within the same country [14]. The unevenness of the subnational territorial spread of COVID-19 has been shown to be related to the structure of local economies [15]. Also, Mohanta et al. [16] identified large differences in the efficiency of India’s states & UTs during the COVID-19 crisis in 2021. Then, the use of regional data allows for a more precise analysis of the impact of demographic and socio-economic risk factors on COVID-19 fatalities because the diversity of these fatalities, and the diversity of the economic risk factors that might be influencing them, is much higher at a regional level than at a national level.

The main contribution of this study to the existing literature is the empirical demonstration of the existence of a health system efficiency / resiliency trade-off, that had not been previously documented. The demonstration of this trade-off is important for policymakers as it shows that policies to improve economic efficiency have a significant cost in terms of resilience, and vice-versa. The study also extends the existing (limited) literature about the economic risk factors that influence the fatalities associated with COVID-19, highlighting the economic regional factors that contributed to increase the health costs of COVID-19.

The rest of this paper is organized as follows. Section “Methods” describes the methodology used. Results are presented in Section “Results” and discussed in Section “ Discussion”. Section   “Conclusion” concludes.

## Methods

In this study, Data Envelopment Analysis (DEA) is used to construct an efficiency index for regional health systems. Then, a spatial econometrics model is estimated, with the number of COVID-19 deaths per hundred thousand inhabitants (the proxy for health system resilience) as the dependent variable, and the efficiency index as one of the explanatory variables. All estimations were performed using the software Stata17 [17].

### Health system efficiency index

Economists distinguish between allocative inefficiency – which arises when the wrong mix of services is provided, given societal preferences, or when a suboptimal mix of inputs is used – and technical efficiency, the effectiveness of a given set of inputs to produce a given set of outputs or outcomes [18]. Given that the focus of this study is on the possible need to accumulate unused resources to foster resilience, a technical efficiency index is constructed to measure how effectively healthcare inputs are being used to produce health system outcomes. DEA is used to construct the efficiency index because it is by far the most common method for analyzing efficiency in health care [19].

Several DEA health system efficiency studies may be found in the literature, most of them at the country level (see Varabyova and Müller [20] for a review and Gavurova et al. [21] or Cetin and Bahce [22] for more recent work), but also at a regional level (for example Carrillo and Jorge [23], Chai et al. [24] or Mohanta et al. [16]). These studies use health expenditure and/or measures of labor inputs (number of physicians, nurses, or other personnel) and capital inputs (number of hospital beds or MRIs) as inputs for the DEA model. Given that the focus of this study is on the use of existing resources, the DEA inputs we use are measures of labor and capital inputs, namely the number of medical doctors, the number of nurses, and the number available beds in hospitals (all variables defined as ratios to population). Health expenditure is not added to the list of inputs because expenditure arises from purchases of labor and capital inputs and adding all together as inputs in the DEA model would seem to double count inputs and could invalidate the findings, and also because differences in input costs across countries could lead to erroneous efficiency estimates [25]. Some studies also use as inputs some social (e.g. education levels), economic (e.g. income inequality) or behavioral variables (e.g. tobacco or alcohol consumption), that this study does not include because the focus is on the use of existing healthcare resources. Data availability at a regional level also conditioned the choice of inputs used.

Most DEA health system efficiency studies use population health indicators as outputs in the DEA model. The most common indicators used are Life expectancy and the Infant mortality rate, but other indicators can be found in the literature, such as Healthy life years [26], Potential years of life lost [27], Mortality rate [28], Avoidable mortality [21] or Self-perceived health status [23]. Data availability determined that the population health indicators used as outputs in the efficiency estimation were life expectancy, potential years of life lost and the standardized mortality rate.^1^

The DEA analysis includes two basic conceptual models: the CCR model [29] that assumes constant returns to scale (CRS) and the BCC model [30] that assumes variable returns to scale (VRS). The literature on health system efficiency includes several examples of both approaches. The advantage of VRS is that one can eliminate size differences between countries, but the VRS frontier draws in more hospitals to the frontier, so more are given a score as efficient, making CRS more discriminatory as to efficiency differences [19]. That is the reason why this study uses the CRS model, together with the fact that scale effects are less important when the analysis is performed with the variables in ratio form, as it is the case here.

The literature on health system efficiency is also divided on whether the DEA model should be input- or output-oriented. In input-oriented models efficiency scores measure the reduction in inputs used to achieve given outcomes, while in output-oriented models efficiency scores correspond to the largest feasible proportional expansion in outputs for given inputs [29]. Given that the focus of this study is on the eventual need to accumulate excess inputs to strengthen health system resiliency, an input-oriented DEA model was used.

The DEA model was estimated using the “dea” command [31] in Stata17 [17].

### Spatial econometrics model for COVID-19 deaths

The regional and local impacts of the COVID-19 crisis are highly heterogeneous, with a strong regional dimension that has important consequences for crisis management and policy responses [14]. Differences in the impact of epidemics between regions may arise from two sources. First, the literature highlights the importance for the diffusion of epidemics of socio-economic and demographic risk factors that may differ significantly across regions [32]. Second, there is a geographical dimension in the diffusion of epidemics, with many studies highlighting the effects of spatial dependence between regions, partially explaining the spatial heterogeneity of the spread of epidemics [33].

COVID-19 spreads through contact, so the level of the epidemic and its consequences (namely deaths) in one area is likely to be related to the same variables in neighboring areas. To incorporate the spatial dependence between areas in the COVID-19 death rate, spatial lag models, also known as spatial autoregressive (SAR) models, were estimated, using COVID-19 deaths as the dependent variable. SAR models incorporate endogenous interaction effects, in which the value of the dependent variable for one area is jointly determined with that of neighboring areas [34]. SARAR models, which consider spatial interaction effects among the error terms in addition to the interaction effects among the dependent variable were also considered. The existence of spatial interaction effects among the error terms is consistent with a situation where determinants of the dependent variable omitted from the model are spatially autocorrelated, or with a situation where unobserved shocks follow a spatial pattern [34]. The SAR model is described by Eq. 1 with *μ* = *ε*, and the SARAR model by Eqs. 1 and 2:

1 $${Y}=\delta WY+X\beta +\mu$$

2 $$\mu =\lambda W\mu +\varepsilon$$

where the vector *Y* corresponds to the number of COVID-19 deaths per hundred thousand inhabitants for each area *i*. *W* is a matrix of spatial weights, *δ* is the spatial autoregressive coefficient and *λ* is the spatial autocorrelation coefficient. *X* is a vector of explanatory variables and *β* is a vector of parameters to be estimated. Finally, $\varepsilon$ is a perturbation term that follows the standard assumptions.

The spatial weights matrix *W* used in this study is a contiguity matrix since it is assumed that COVID-19 spreads between adjacent areas. Thus, the generic element *W*_*ij*_ of the matrix linking area *i* and area *j* is 1, if the two areas share a border, and 0 otherwise. The matrix ***W*** was created by applying the “spmatrix” command in Stata 17 [17] to the European NUTS 2016 shapefile (see Section  “Data”).

The explanatory variables vector ***X*** includes the health system efficiency index computed as described above, and other variables that have been identified as significant determinants of COVID-19 deaths in previous literature (namely in [14, 32, 35–38]), that can be classified in four groups.

The first group of explanatory variables measures demographic characteristics of the population. COVID-19 deaths were more frequent for older people, so the age structure of the population is a variable used in all the studies cited above; different indicators of the age structure were used in these studies, such as the percentage of the population aged 65 (or 60 or 75 or 85) years or over, or the average (or median) age. Ehlert [32] showed that the sex structure of the population, measured by the share of women, may also be a factor affecting COVID-19 deaths. Another demographic variable frequently used in these studies is an indicator of the level of concentration in the distribution of the population, since COVID-19 spreads more easily in areas with a high concentration of people, where frequent contacts with many persons are more common; this indicator is usually the population density but may also be some measure of urbanization [14, 32, 36].

The second group relates to economic factors. Income levels have been related to health outcomes, so all those studies use some indicator related to the income level (such as Gross Domestic Product per capita at purchasing power parities or Household income) or some variable related to material deprivation, such as the poverty rate, the unemployment rate, or a measure of inequality (Gini coefficient). The other economic factor found to be related to COVID-19 deaths is some measure of the economic structure of the region, since some workers are at higher risk of COVID-19 than others, because they work in physical proximity to other people (such as in manufacturing), or because they are more exposed to international contacts (in the tourism sector), or are at lower risk of COVID-19 because they are more able to work remotely (as it is the case for some services); the share of employees in the industry sector [35] or in the service sector [32], and the number of inbound tourists [38] have been used as measures of the exposure to COVID-19 due to the economic structure of the region.

The third group of variables measures social characteristics, such as social capital, education attainment (percent of the population with secondary/tertiary education), housing and living conditions (household size, multigenerational households with older people, nursing homes), and migration (net migration or the share of foreigners in the population). Due to data availability, only an indicator measuring the share of the adult population with at least secondary education is used in this study.

Finally, a fourth group of variables are related to resources and outcomes of healthcare services: input variables such as the number of physicians and hospital beds; indicators of the population health status, such as life expectancy or the prevalence of specific diseases. Since the health system efficiency index is a measure computed from these health system variables, adding other healthcare sector variables would imply some duplication of information and possibly some strong collinearities. Furthermore, the health system efficiency index summarizes the level of healthcare inputs used in relation to the health outcomes. As such, the only variable included in the model related to the healthcare sector is the health system efficiency index.

The spatial econometrics models were estimated using the “spregress” command in Stata 17 [17], with a generalized spatial two-stage least-squares estimator, which is more robust than a maximum-likelihood estimator, since the former assumes that the errors are independent and identically distributed but does not require normality.

### Data

This study covers the 27 countries in the European Union and the 4 countries in the European Free Trade Association. NUTS-2 is the preferred regional level of analysis since NUTS-2 represents the basic region for the application of regional policies.^2^ However, for some countries crucial data (namely COVID-19 deaths) were only available at NUTS-1 level (for Belgium, Croatia, Germany, Ireland, France, Lithuania, Slovenia, and Slovakia) or at NUTS-0 level (for Bulgaria, Greece, Hungary, and Finland); for those countries, NUTS-1 or NUTS-0 level regions, respectively, were considered and pooled with the NUTS-2 regions for other countries, generating a sample of 173 areas.

**Fig. 1:**
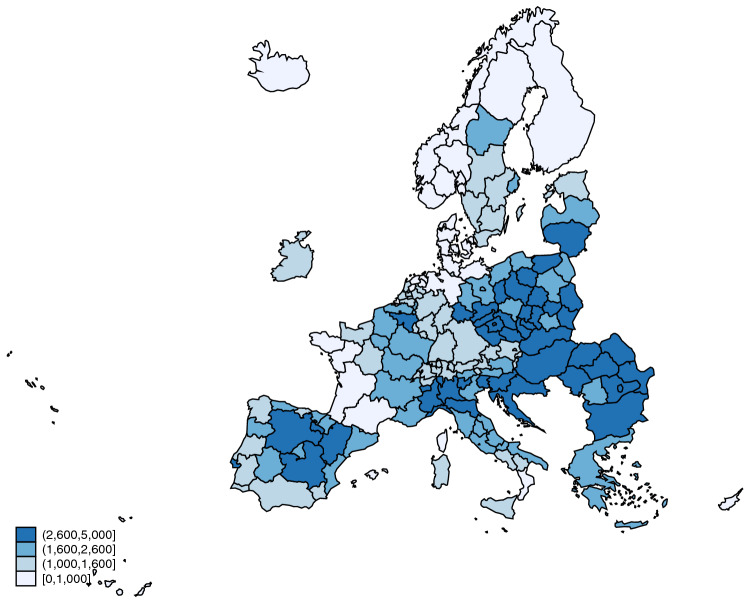
COVID-19 death rates by region. The figure represents the number of COVID-19 deaths (up to December 31, 2021) per million inhabitants, by region

Geographic data for the regions in the sample were extracted from the NUTS 2016 shapefile available at Eurostat, the official European Union statistics database, which was the source for most of the data used. Data regarding the number of COVID-19 deaths refer to the cumulative number of deceased up to December 31st, 2021, and were (mostly) obtained from The Humanitarian Data Exchange website, managed by OCHA – United Nations Office for the Coordination of Humanitarian Affairs. Figure 1 provides a map with the COVID-19 death rate (number of COVID-19 deaths per million inhabitants) for each area. A full description of data sources is provided in Appendix 1.

The latest data available in the Eurostat database for potential years of life lost (PYLL) and the standardized death rate by NUTS2 regions are from 2017, and are computed as 3-year averages, implying that these data are affected by deaths that occurred in 2015. For this reason, in the DEA efficiency estimation model, the outputs data refers to the year 2017 and the inputs data refer to the year 2015, since the healthcare resources available in 2015 were the resources that were most relevant in determining the deaths occurred in 2015 (and were still relevant for the deaths occurring in 2016 and 2017). All inputs data refer to numbers per 100,000 inhabitants. Table 1 provides summary statistics for the variables used to estimate the health system efficiency index.

**Table 1:** Summary statistics for the variables used in the DEA model

Variable	Mean	Median	Standard deviation	Minimum	Maximum
Inputs (2015)					
Medical doctors	368	364	99	129	712
Nurses	972	821	435	345	2,070
Hospital beds	507	482	221	160	1,302
Outputs (2017)					
Life expectancy	81.2	81.9	2.5	74.5	85.1
PYLL	3,492	3,015	1,238	2,157	7,392
Standardized death rate	1,017	969	201	730	1,628

*PYLL* potential years of life lost. All variables refer to ratios per 100,000 inhabitants, except Life expectancy (in years)

The DEA efficiency estimation method assumes that efficiency is increased when outputs are increased for a given set of inputs. Mortality indicators, such as potential years of life lost or standardized mortality rates, decrease when the health of the population increases, implying that these indicators must be transformed before use as DEA outputs. Following Afonso and St. Aubyn [27] and Sun et al. [39], the indicators used are Potential Years of Life Not Lost (PYLNL) and the Standardized Survival Rate (SSR), defined as:

SSR = ln(100,000) – ln(standardized death rate);

PYLNL = ln(3,750,000) – ln(PYLL), where PYLL refers to Potential Years of Life Lost per 100,000 population under 75 years and 3,750,000 is an estimate of the number of potential years of life lost per 100,000 population under 75 years, if mortality was random.

The spatial econometrics model uses the COVID-19 death rate for 2020 and 2021 as the dependent variable, while data for the explanatory variables refer to 2019 (or to January 1st, 2020, in case of variables related to population numbers), to avoid possible endogeneities (some of the variables were affected by COVID-19) and to have a more precise characterization of the regional risk factors at the start of the COVID-19 pandemic. The indicators used as explanatory variables are as follows:

- Age structure: percentage of the population aged 65 years or older;
- ShareW: percentage of women in total population;
- Density: population density (persons by square Kilometer);
- Poverty: percentage of people at risk of poverty or social exclusion;
- Industry: percentage of employment in industry sectors (NACE Rev. 2 sectors B-F) in total employment;
- Education: percentage of 25–64-year-olds with upper secondary, post-secondary non-tertiary and tertiary education (levels 3–8).

Table 2 provides summary statistics for the variables used in the spatial econometrics model.

**Table 2:** Summary statistics for the variables used in the spatial econometrics model

Variable	Mean	Median	Standard deviation	Minimum	Maximum
COVID-19 death rate	1,823	1,610	980	102	4,501
Age structure	20.2	20.0	3.0	13.1	28.6
ShareW	50.89	50.91	0.89	48.35	53.82
Density	332	118	816	3	7,552
Poverty	20.2	18.1	7.7	7.9	49.7
Industry	24.2	23.4	7.9	9.9	48.5
Education	78.6	82.1	13.0	33.5	97.2

*ShareW* share of women in the population

## Results

**Fig. 2:**
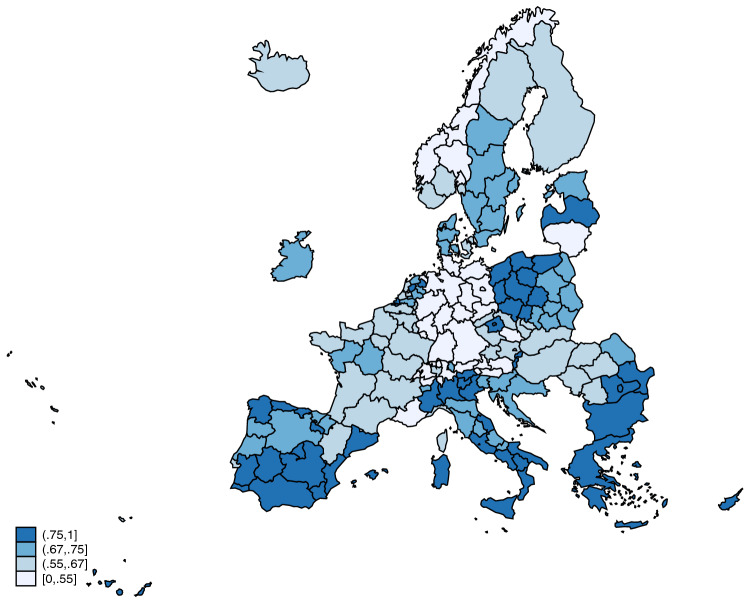
Health system efficiency by region. The figure represents the value of the health system efficiency index, by region, estimated using DEA

The health system efficiency index was estimated using a DEA model with the number of medical doctors, nurses, and beds, per 100,000 inhabitants, as inputs, and life expectancy, the standardized survival rate, and potential years of life not lost as outputs. The mean value for the efficiency index is 0.669 and the median value is 0.676. The efficient frontier includes the areas of Greece, Andalucia (Spain), Liechtenstein, Flevoland (Netherlands), and Wielkopolskie (Poland). The less efficient area is Praha (Czechia), with an efficiency index of 0.354, followed by the German states of Hamburg, Mecklenburg-Vorpommern, Bremen, Saarland, and Thüringen. Figure 2 provides a map with the efficiency level for each area.

The second step in this study is the estimation of a model explaining the regional differences in the COVID-19 death rate, including the health system efficiency index estimated in the first step as one of the explanatory variables. Estimating a linear regression using Ordinary Least Squares (OLS) is not an adequate methodology, because the error terms in that regression exhibit spatial dependence. Applying the Moran test for spatial dependence to the error terms of an OLS regression, using a contiguity matrix, delivers a Chi^2^(1) = 31.05 (*p*-value 0.000). This result validates the option for a spatial econometrics model as described in Sect. “Spatial econometrics model for COVID-19 deaths” above.

Table 3 provides the estimation results for the spatial autoregressive SAR model explaining the regional distribution of COVID-19 death rates (C19deaths). Column (1) presents the coefficient estimates (and respective p-values) for the SAR model. In SAR models, the existence of spatial spillover effects implies that the estimation coefficients do not measure the effects of the associated explanatory variable on the dependent variable but are ingredients to a recursive calculation of those effects. The result of that recursive process is the average impact of each variable on the COVID-19 death rate, presented in columns (2) to (4).

**Table 3:** Estimation results for the SAR model, with COVID death rate as dependent variable

Variable	Coefficient estimates (*p*-values)	Average impacts:			Increase in C19deaths
	(1)	Direct (2)	Indirect (3)	Total (4)	(median to maximum) (5)
Efficiency	1280.9 *** (0.001)	1305.8 *** (0.001)	498.7 ** (0.015)	1804.5 *** (0.002)	585
Age structure	25.778 (0.150)	26.278 (0.150)	10.037 (0.174)	36.316 (0.150)	312
ShareW	215.95 *** (0.000)	220.14 *** (0.000)	84.085 *** (0.005)	304.23 *** (0.000)	885
Population density	0.2987 *** (0.000)	0.3045 *** (0.000)	0.1163 *** (0.007)	0.4209 *** (0.000)	3129
Poverty	14.088 * (0.053)	14.362 * (0.053)	5.485 * (0.092)	19.847 * (0.057)	627
Industry	44.089 *** (0.000)	44.945 *** (0.000)	17.167 *** (0.000)	62.112 *** (0.000)	1559
Education	8.8486 * (0.082)	9.0204 * (0.082)	3.4454 (0.104)	12.4658 * (0.081)	188
Constant	− 13,218.2 *** (0.000)				
*ρ*	0.3854 *** (0.000)				
Pseudo R2	0.5250				

*P* values in parenthesis. ***significant at the 1% level; **significant at the 5% level; *significant at the 10% level

Table 4 provides the estimation results for the equivalent SARAR model explaining the regional distribution of COVID-19 death rates (C19deaths). The SARAR model considers spatial interaction effects among the error terms in addition to the interaction effects among the dependent variable that were considered for the SAR model in Table 3. Column (1) presents the coefficient estimates (and respective p-values) for the SARAR model. The significance of the estimate for the parameter λ suggests that interaction effects among the error terms are relevant. The average impact of each variable on the COVID-19 death rate is presented in columns (2) to (4). Column (5) of Table 3, 4 provides estimates of the difference in C19 deaths between a region with median level of the explanatory variable, and a region with the maximum value for that variable, all other things equal (equal to the estimate of total impact for that variable in column 4 times the difference between the maximum and the median values for that variable).

**Table 4:** Estimation results for the SARAR model, with COVID death rate as dependent variable

Variable	Coefficient estimates (*p*-values)	Average impacts:			Increase in C19deaths
	(1)	Direct (2)	Indirect (3)	Total (4)	(median to maximum) (5)
Efficiency	952.72 ** (0.023)	969.09 ** (0.023)	341.98 * (0.053)	1311.08 ** (0.024)	425
Age structure	27.756 (0.133)	28.232 (0.133)	9.963 (0.166)	38.195 (0.135)	328
ShareW	220. 5 *** (0.000)	224.28 *** (0.000)	79.148 ** (0.011)	303.43 *** (0.000)	883
Population density	0.2738 *** (0.000)	0.2786 *** (0.000)	0.0983 ** (0.014)	0.3769 *** (0.000)	2802
Poverty	11.39 (0.145)	11.585 (0.145)	4.088 (0.177)	15.674 (0.146)	495
Industry	39.132 *** (0.000)	39.805 *** (0.000)	14.047 *** (0.000)	53.851 *** (0.000)	1352
Education	10.1761 * (0.07)	10.351 * (0.069)	3.652 * (0.087)	14.004 * (0.066)	211
Constant	− 13,136.5 *** (0.000)				
*ρ*	0.3649 *** (0.000)				
*λ*	0.5204 *** (0.003)				
Pseudo *R*2	0.5048				

*P*-values in parenthesis. ***significant at the 1% level; **significant at the 5% level; *significant at the 10% level

The results in Tables 3 and 4 show that there is significant spatial dependence in the dependent variable COVID-19 death rate and in the error term since the estimates for the spatial autoregressive coefficient *δ* and the spatial autocorrelation coefficient *λ* are both statistically significant at the 1% level. The pseudo R2 above 0.5 and the significance of most of the variables in the model confirm that regional differences in COVID-19 deaths can be partially explained by demographic, economic, social and health system-related factors.

Three variables are significant at the 1% level in both models: population density, the share of industry sector employment in total employment and the share of women in the population. The magnitude of the effect of the population density is roughly double the effect of the industry employment and more than three times the effect of the share of women. The effect of the population age structure, measured by the percentage of the population aged 65 years or older, is not significant, but a significant effect of the population sex structure, measured by the share of women in the population, was found. The education indicator is significant at the 10% level in both models, but the magnitude of the effect is relatively small, even smaller than the effect of the age structure, which is not statistically significant. The poverty rate has a slightly larger effect, but it is only statistically significant (at the 10% level) in the SAR model.

The main result of the model is the significant impact of the health system efficiency index on C19deaths (at the 1% level in the SAR model and at the 5% level in the SARAR model). The magnitude of the total impact estimated by the SAR model implies that a region in the efficiency frontier will have more 585 deaths per million inhabitants (36% of the median of C19deaths) than a region with median efficiency. The magnitude of the impact estimated by the SARAR model is smaller, but still represents 425 deaths per million inhabitants (26% of the median of C19deaths). Both models show that health system efficiency had a significant and relevant impact on COVID-19 deaths: the regions with more efficient health systems before the pandemic were the regions with higher COVID-19 death rates.

### Robustness checks

Several alternative estimations were performed to assess the robustness of the results for the impact of the health system efficiency index on C19deaths. Appendix 2 summarizes the results for the estimation of the alternative models used for robustness checks and shows that the total impact of the efficiency index on COVID-19 death rate, and its level of significance, are robust across different model specifications.

First, the model was estimated using different measures of efficiency, obtained using different DEA estimation methods – namely, using variable returns to scale or using an output-oriented DEA – or using different combinations of outputs: without PYLNL, without SSR, or adding the infant survival rate (the inverse of the infant mortality rate). The estimate for the total impact of the efficiency index on COVID-19 death rate was very similar for these five alternative models, ranging from 1132.1 to 1349.3 (with p-values ranging from 0.008 to 0.049) for the SARAR model, and ranging from 1613.6 to 1838.5 (with p-values ranging from 0.001 to 0.004), for the SAR model.

Second, the model was estimated using different indicators for the explanatory variables. For the age structure of the population, the alternative measures used (instead of the percentage of the population aged 65 years or older) were: the percentage of the population aged 75 years or older; the percentage of the population aged 85 years or older; the median age. One model was estimated without the variable percentage of women in total population. One model used the Gross domestic product at purchasing power standard per capita (instead of the Poverty rate). The economic structure of the region was measured, in alternative to the share of employees in Industry, by the share of employees in the Service sector or by the number of arrivals at Tourist accommodation establishments per inhabitant. The estimate of the total impact of the efficiency index on COVID-19 death rate was of the same order of magnitude in these seven alternative models, ranging from 1180.7 to 1761.2 (with p-values ranging from 0.010 to 0.038) for the SARAR model, and ranging from 1660.8 to 2663.8 (with p-values ranging from 0.000 to 0.005) for the SAR model.

Finally, three other estimations were performed: (i) an inverse distance spatial weights matrix was used instead of a contiguity spatial weights matrix; (ii) the spatial econometrics models were estimated without Liechtenstein, since the comparability of the data for the economic and education variables for Liechtenstein could be questioned since they were obtained from different sources and referred to earlier years; and (iii) an “estimated excess mortality rate”^3^ was used as dependent variable instead of C19deaths. In these three estimations the effect of the efficiency index on the dependent variable was stronger than in the models in Tables 3 and 4, with higher estimates for the coefficient and for the total impact of efficiency.

## Discussion

COVID-19 had a significant impact on mortality across Europe, but there were significant differences across countries, and even across regions of the same country. The nature of the disease, a respiratory disease that is transmitted by contact with infected persons, suggested that strong spatial dependence might be found in COVID-19 deaths, which was confirmed by our results showing significant estimates for the spatial dependence coefficients associated with the dependent variable COVID-19 death rate and the error term.

The main result of this study is the estimation of a significant and large effect of health system efficiency on COVID-19 death rates. More efficient health systems, i.e., systems that use less inputs to achieve a given level of health outcomes, have higher COVID-19 death rates. The effect of efficiency on the response to the COVID19 pandemic on each region may result from three different pathways. First, since more efficiency implies lower levels of resources for a given level of outcomes, then more efficient regions are likely to have lower surge capacity, i.e., high efficiency regions had less resources available to accommodate the surge of patients that occurred during the COVID19 pandemic. Second, it is likely that the willingness to spend resources on items that have a low immediate impact is lower in regions where efficiency is higher on the priority list. Health system decision makers driven by efficiency concerns might reduce spending on resilience building investments – such as preparing and implementing crisis contingency plans, reinforcing crucial infrastructure, or buying masks and other protective equipment – because these expenditures may seem unjustifiably costly when a pandemic is not a concern. That would imply that more efficient regions were less prepared to deal with COVID19 when the pandemic was declared. Third, efficiency usually implies streamlining and specialization, which could imply that more efficient regions had lower flexibility and less redundancies that were crucial during the COVID19 pandemic. For example, more efficient regions might have only one hospital where less efficient ones had two or three; when COVID19 spread across the staff of one hospital, the latter would still have other hospitals operational to respond to patients while the former would not be able to provide care.

The significant and large effect of health system efficiency on COVID-19 deaths identified suggests that a health policy trade-off may exist between health system efficiency and health system resilience. Due to limitations on the data available, this study only analyzed European countries during the COVID19 pandemic and used a measure of health system resilience (COVID19 deaths) that may not be the most appropriate (a measure of excess mortality would be preferable), but nevertheless the results on the efficiency effect were robust across several different models. As more data becomes available, future work might analyze other regions of the world, during COVID19 and other health crises, and using alternative measures of health system resilience. If the efficiency / resilience trade-off identified in this study is found to be a general feature of health systems, then when policymakers focus on health system efficiency, the resilience of the health system decreases, and the health costs of the next health crisis will be higher; when policymakers invest in health system resilience, the current costs of the health system increase and efficiency decreases.

The existence of an efficiency / resilience trade-off will imply a choice between present and future costs: a more efficient health system has lower costs in non-crisis periods but will suffer higher costs when a health crisis arrives. Increasing health system resilience thus implies using more resources, that are diverted from other policies (e.g., education, social safety nets) and will not be efficiently used in the health sector. Then, policy prescriptions to strengthen resilience of health systems cannot be based only on the observation of the health costs associated with the COVID-19 pandemic but need to be weighed against the costs of the inefficiency that increasing resilience will generate.

This study also found that demographic, economic and social factors explain part of the regional differences in COVID-19 deaths, in line with previous literature. In particular, it was found that variables that reflect more proximity with other people, such as population density and the share of employment in industry, are associated with higher COVID-19 deaths. A significant effect of population density on C19deaths had already been identified by Ehlert [32], and the large effect found here is likely to reflect the transmission mechanism of COVID-19: in more densely populated areas, such as urban areas, the risk of infection is higher not only because the frequency of contacts is higher but also because the use of public transport may facilitate contagion.

The effect of the age structure, measured by the percentage of the population aged 65 years or older, is not significant, contrary to what was found previously [14, 32, 37]. We did find a significant positive effect of the share of women in the population that might be capturing the same demographic structure that is relevant for C19deaths. Women have a higher life expectancy, so the share of women in older age groups is higher than the share of women in the total population. As such, the significant effect of the age structure found in previous work could be capturing the effect of the share of women we found in this study (this conclusion is reinforced by the significance of the variable Age structure in models estimated without the variable ShareW – see Appendix 2).

The large and significant effect of the share of industry sector employment in total employment may reflect the higher contagion risk associated with manufacturing and construction activities, because in these activities workers have physical proximity to other workers, preventive measures such as remote working, social distancing, and mask wearing were more difficult to implement, and manufacturing activities were less likely than services to be shut down during lockdown periods. Ascani et al. [15] reached a similar conclusion, arguing that manufacturing workers are subject to long shifts and interactions at close distance with a relatively large number of coworkers, as compared to many other occupations, and that it is also plausible that trading manufacturing output requires more human interaction than trading services, as the former consist in most cases of tangible goods that need to be physically shipped.

The education indicator is significant at the 10% level in both models. The magnitude of the effect of education on COVID-19 deaths is relatively small, but it is surprisingly positive. Hawkins [37] found that US counties with a higher proportion of adults with a high school degree had lower fatality rates, which they argued could be explained by the highest paying jobs being more amenable to remote working. The result in this study goes in the opposite direction, probably because the effect of the job structure distribution is being captured by the industry sector employment indicator (and maybe also by the poverty rate). After removing the job structure effect, education might influence mortality through different levels of adherence to protective measures (e.g. mask wearing).

The poverty rate has a slightly larger effect than the education indicator, but it is only statistically significant (at the 10% level) in the SAR model. Ginsburgh et al. [36] found that income inequality is positively related to COVID-19 deaths, and hypothesize that poorer individuals are more likely to have pre-existing conditions that are known comorbidities or aggravating factors for the course of COVID-19, such as diabetes, obesity, or cardio-vascular diseases, and that poorer individuals are also more likely to be in jobs that cannot be safely distanced through tele-working, such as manufacturing, transportation and distribution, retail, etc.

## Conclusion

This study investigates the existence of a trade-off between health system resilience and efficiency during the COVID19 pandemic, using data for 173 regions in the European Union and the European Free Trade Association countries.

The main result is that regions with higher values of the health system efficiency index in 2017 had significantly higher rates of COVID-19 deaths in 2020 and 2021, indicating a trade-off between the economic efficiency and the resiliency of the health system during the COVID-19 pandemic. The results also show that some economic factors did have a significant effect on COVID-19 deaths: the economic structure, measured by the share of industry sector employment, was associated with a large and significant effect on COVID-19 death rate, and the poverty rate also had some impact, although smaller and less significant.

The results suggest the existence of a health system efficiency / resilience trade-off, which would imply that policy prescriptions to strengthen the resilience of health systems should not be based only on the observation of the health costs associated with the COVID-19 pandemic but need to be weighed against the costs of the inefficiency that increasing resilience will generate.

## Data Availability

The original data on which this study is supported are publicly available at the data sources indicated in Appendix 1. The data used in the estimations that support the findings of this study are openly available in Mendeley Data, with the reference: Almeida, Alvaro (2022), “Data for “Health system resilience in European regions”, Mendeley Data, V1, doi: 10.17632/msppyzbp8c.1.

## Appendix 1: Data description and sources

A. COVID-19 death rate: number of COVID-19 deaths up to December 31st, 2021 per million inhabitants = C19deaths / POP2020 *1,000,000.

where POP2020 is Total population on 1 January 2020 and.

C19deaths is the cumulative number of COVID-19 deaths up to and including December 31st, 2021. If data for December 31st were not available, data were estimated through linear interpolation using the previous and following closest data points available.

Regional C19deaths data were obtained from The Humanitarian Data Exchange website, managed by OCHA – United Nations Office for the Coordination of Humanitarian Affairs, available at https://data.humdata.org/dataset/europe-covid-19-subnational-cases?

For Bulgaria, Estonia, Croatia, and Finland, C19deaths data were obtained from the World Health Organization’s WHO Coronavirus (COVID-19) Dashboard, available at https://covid19.who.int/table.

For some countries, regional C19deaths data were obtained from national sources, namely:

Czechia: https://onemocneni-aktualne.mzcr.cz/covid-19

Denmark: https://experience.arcgis.com/experience/aa41b29149f24e20a4007a0c4e13db1d/page/Regionalt/

France: https://geodes.santepubliquefrance.fr/#c=indicator&f=0&i=covid_hospit.dc&s=2022-02-06&t=a01&view=map1

Norway:

https://www.fhi.no/en/publ/2020/weekly-reports-for-coronavirus-og-covid-19/

Poland: https://www.statista.com/statistics/1102376/poland-coronavirus-covid-19-new-cases-by-region/

Portugal: https://covid19.min-saude.pt/

Romania: https://covid19.geo-spatial.org/

Sweden: https://experience.arcgis.com/experience/2dc63e26f509468f896ec69476b0dab3

B. OTHER DATA: the main source for other data is the database of Eurostat, the statistical office of the European Union, available at https://ec.europa.eu/eurostat/data/database. The variable definitions and the specific datasets from where the data were extracted were:

- *Medical doctors*: number of medical doctors per hundred thousand inhabitants; dataset “Health personnel by NUTS 2 regions”.
- *Nurses*: number of nurses and midwives per hundred thousand inhabitants; dataset “Health personnel by NUTS 2 regions”.
- *Hospital beds*: number of available beds in hospitals per hundred thousand inhabitants; dataset “Hospital beds by NUTS 2 regions”.
- *Life expectancy*: at age less than 1 year; dataset “Life expectancy by age, sex and NUTS 2 region”.
- *Potential years of life lost*: all causes of death (A00-Y89) excluding S00-T98; dataset “Causes of death—years and potential years of life lost by NUTS 2 regions of residence, 3-year average”.
- *Standardized death rate*: all causes of death (A00-Y89) excluding S00-T98; dataset “Causes of death—standardized death rate by NUTS 2 region of residence, 3 year average” (per 100,000 inhabitants).
- *ShareW*: percentage of women in total population = Number of females / Total number of population; dataset “Population on 1 January by age group, sex and NUTS 2 region”.
- *Density*: population density = Total number of population / Area in square Kilometers; datasets “Population on 1 January by age group, sex and NUTS 2 region” and “Area by NUTS 3 region”.
- *Age*: Proportion of population aged 65/75/85 years and more; Median age of population; dataset “Population structure indicators by NUTS 2 region”.
- *Poverty*: percentage; dataset “People at risk of poverty or social exclusion by NUTS regions”.
- *Industry*: share of employees in industry = ((Employment in Industry except construction B-E) + (Employment in Construction F)) / (Total employment—all NACE activities) × 100; dataset “Employment by sex, age, economic activity and NUTS 2 regions”.
- *Education*: percentage of population from 25 to 64 years with upper secondary, post-secondary non-tertiary and tertiary education (levels 3–8); dataset “Population by educational attainment level, sex and NUTS 2 regions (%)”.
- **GEOGRAPHIC** information for the regions in the sample was provided by the NUTS 2016 shapefile (NUTS_RG_20M_2016_3035) available at https://ec.europa.eu/eurostat/web/gisco/geodata/reference-data/administrative-units-statistical-units/nuts
- Other data sources used were:
- Belgium, *Nurses*: OECD Health Statistics 2021, available at https://www.oecd.org/health/health-data.htm
- Liechtenstein, *Poverty*: data from a survey by the Liechtenstein’s Office for Social Services reported in.
- https://ec.europa.eu/social/BlobServlet?docId=16902&langId=en
- Liechtenstein, *Industry*: data from CIA World Factbook, available at.
- https://www.cia.gov/the-world-factbook/countries/liechtenstein/
- Liechtenstein, *Education*: at least completed upper secondary, population 25 + , total (%) (cumulative), available at the World Bank’s World Development Indicators,
- https://databank.worldbank.org/source/world-development-indicators#
- Data for *Nurses* for the Belgium, Germany, and Switzerland regions, and data for *Poverty* for the regions of France, were obtained by applying the regional distribution obtained from national sources to the national numbers from Eurostat. The national sources were:
- Belgium, *nurses*: “nombre d’infirmiers par région de travail”, available in “Personnel des soins de santé par Région de travail”, https://statbel.fgov.be/fr/themes/datalab/personnel-des-soins-de-sante#figures.
- Germany, *nurses*: distribution of total non-medical staff, data for hospitals available at https://www.destatis.de/EN/Themes/Society-Environment/Health/Hospitals/Tables/staff-hospitals-laender.html;jsessionid=B64B7C1A728AE4497DE8575603580799.live742
- and data for other healthcare services available at https://www.gbe-bund.de/gbe/trecherche.prc_them_rech?tk=14501&tk2=15352&p_uid=gast&p_aid=51949665&p_sprache=D&cnt_ut=17&ut=15352
- Switzerland, *nurses*: distribution of total health staff, calculated as the sum of “Personnel hospitalier”, “Personnel des établissements médico-sociaux”, and “Personnel des services d'aide et de soins à domicile”, available at https://www.atlas.bfs.admin.ch/maps/13/fr/12370_7287_4422_7264/20395.html
- France, *poverty*: “Taux de pauvreté selon l'âge du référent fiscal en 2019: comparaisons regionals”, available at https://www.insee.fr/fr/statistiques/2012803#tableau-TCRD_024_tab1_regions2016

## Data availability

The data used in the estimations are available at: Almeida, Alvaro (2022), “Data for “Health system resilience in European regions””, Mendeley Data, V1, doi: 10.17632/msppyzbp8c.1.

## SAR models estimated using alternative efficiency measures (health system efficiency estimated using alternative DEA models)

**Table:**

Variable	Model 1 (variable returns to scale)	Model 2 (output-oriented DEA)	Model 3 (without PYLNL)	Model 4 (without SSR)	Model 5 (with Infant Survival rate)
Efficiency	1174.8*** (0.001)	1280.9*** (0.001)	1267.2*** (0.002)	1301.8*** (0.001)	1177.8*** (0.003)
Population density	0.3030*** (0.000)	0.2987*** (0.000)	0.2979*** (0.000)	0.2996*** (0.000)	0.2949*** (0.000)
Age structure	22.412 (0.203)	25.778 (0.150)	25.511 (0.154)	26.234 (0.143)	24.233 (0.176)
ShareW	208.01*** (0.000)	215.95*** (0.000)	215.68*** (0.000)	216.89*** (0.000)	212.41*** (0.000)
Industry	48.516*** (0.000)	44.089*** (0.000)	44.186 *** (0.000)	43.709*** (0.000)	44.519*** (0.000)
Poverty	17.371** (0.017)	14.088* (0.053)	14.042* (0.054)	13.938* (0.055)	14.066 * (0.055)
Education	9.3073* (0.066)	8.8486* (0.082)	8.7966* (0.084)	8.8788* (0.080)	8.1798 (0.108)
Constant	− 12,936.1*** (0.000)	− 13,218.2*** (0.000)	− 13,184.6 *** (0.000)	− 13,278.3 *** (0.000)	− 12,899.7*** (0.000)
*ρ*	0.3631*** (0.000)	0.3854*** (0.000)	0.3847*** (0.000)	0.3876*** (0.000)	0.3852*** (0.000)
Pseudo *R*2	0.5275	0.5250	0.5239	0.5261	0.5207
Efficiency total impact	1613.6*** (0.001)	1804.5*** (0.002)	1783.7*** (0.002)	1838.5*** (0.002)	1658.8*** (0.004)

Each column presents the coefficient estimates (and respective p-values in parenthesis) for a SAR model estimated using a different measure of efficiency. The final line presents the estimate for the total impact on the dependent variable C19deaths. ***significant at the 1% level; ** significant at the 5% level; * significant at the 10% level

## SAR models estimated using alternative combinations of explanatory variables

**Table:**

Variable	Model 6	Model 7	Model 8	Model 9	Model 10	Model 11	Model 12
Efficiency	1260.8*** (0.002)	1186.2*** (0.003)	1276.2*** (0.002)	1373.9*** (0.001)	1311.9 *** (0.001)	1187.4*** (0.004)	1593.1*** (0.000)
Population density	0.2854*** (0.000)	0.2810*** (0.000)	0.2969*** (0.000)	0.3306*** (0.000)	0.3124*** (0.000)	0.3307*** (0.000)	0.2312*** (0.001)
Age structure	23.815^a^ (0.355)	49.364^b^ (0.492)	22.762^c^ (0.213)	37.825** (0.038)	18.111 (0.259)	34.865* (0.054)	32.568* (0.089)
ShareW	220.60*** (0.000)	221.33*** (0.000)	216.67*** (0.000)		229.85 *** (0.000)	229.74 *** (0.000)	240.44 *** (0.000)
Industry	45.048*** (0.000)	45.593*** (0.000)	43.991*** (0.000)	45.332*** (0.000)	43.244*** (0.000)		
Services (share)						-31.279*** (0.000)	
Tourism							-11.385 (0.624)
Poverty	13.419* (0.066)	13.774* (0.064)	13.604* (0.061)	18.134** (0.015)		1.634 (0.833)	10.283 (0.217)
GDP PPPpc					-0.0013 (0.784)		
Education	8.5291 (0.108)	8.3706 (0.131)	9.1366* (0.083)	8.2040 (0.119)	5.4287 (0.261)	8.7527 * (0.092)	15.556 *** (0.005)
Constant	− 13,120 *** (0.000)	− 13,023 *** (0.000)	− 13,724 *** (0.000)	− 2660*** (0.001)	− 13,212 *** (0.000)	− 10,594 *** (0.000)	− 14,316 *** (0.000)
*ρ*	0.3806*** (0.000)	0.3800*** (0.000)	0.3783*** (0.000)	0.4256*** (0.000)	0.3745*** (0.000)	0.4215*** (0.000)	0.5165*** (0.000)
Pseudo *R*2	0.5217	0.5208	0.5235	0.4759	0.5113	0.5052	0.3650
Efficiency impact	1766.2*** (0.003)	1660.8*** (0.003)	1783.2*** (0.002)	2031.2*** (0.001)	1825.3*** (0.002)	1746.5*** (0.005)	2663.8*** (0.000)

Each column presents the coefficient estimates (and respective p-values in parenthesis) for a given SAR model

^a^Age structure indicator: percentage of the population aged 75 years or older

^b^Age structure indicator: percentage of the population aged 85 years or older

^c^Age structure indicator: median age of the population

***significant at the 1% level; **significant at the 5% level; *significant at the 10% level

## SARAR models estimated using alternative efficiency measures (health system efficiency estimated using alternative DEA models)

**Table:**

Variable	Model 1 (variable returns to scale)	Model 2 (output-oriented DEA)	Model 3 (without PYLNL)	Model 4 (without SSR)	Model 5 (with Infant Survival rate)
Efficiency	945.52*** (0.008)	952.72** (0.023)	939.72** (0.025)	977.75** (0.020)	823.44** (0.048)
Population density	0.2810*** (0.000)	0.2739*** (0.000)	0.2728*** (0.000)	0.2753*** (0.000)	0.2682*** (0.000)
Age structure	28.201 (0.124)	27.756 (0.133)	27.614 (0.135)	28.074 (0.129)	26.558 (0.152)
ShareW	214.81*** (0.000)	220.5*** (0.000)	220.3*** (0.000)	221.0*** (0.000)	217.0*** (0.000)
Industry	40.990*** (0.000)	39.132*** (0.000)	39.120*** (0.000)	39.193*** (0.000)	39.152*** (0.000)
Poverty	13.105* (0.095)	11.390 (0.145)	11.325 (0.148)	11.347 (0.146)	11.299 (0.121)
Education	10.919** (0.049)	10.1761* (0.07)	10.1761* (0.07)	10.206* (0.068)	9.446* (0.092)
Constant	− 13,034*** (0.000)	− 13,137*** (0.000)	− 13,109*** (0.000)	− 13,183*** (0.000)	− 12,796*** (0.000)
ρ	0.3522*** (0.000)	0.3649*** (0.000)	0.3639*** (0.000)	0.3673*** (0.000)	0.3640*** (0.000)
λ	0.5542*** (0.001)	0.5204*** (0.003)	0.5254*** (0.003)	0.5134*** (0.003)	0.5333*** (0.002)
Pseudo R2	0.5068	0.5048	0.5035	0.5063	0.4986
Efficiency total impact	1283.1*** (0.008)	1311.1** (0.024)	1291.8** (0.026)	1349.3** (0.021)	1132.1** (0.049)

Each column presents the coefficient estimates (and respective *p*-values in parenthesis) for a SARAR model estimated using a different measure of efficiency. The final line presents the estimate for the total impact on the dependent variable C19deaths. ***significant at the 1% level; **significant at the 5% level; *significant at the 10% level

## SARAR models estimated using alternative combinations of explanatory variables

**Table:**

Variable	Model 6	Model 7	Model 8	Model 9	Model 10	Model 11	Model 12
Efficiency	930.35** (0.029)	865.91** (0.038)	938.99** (0.026)	978.93** (0.024)	948.07** (0.026)	965.20** (0.023)	1146.5** (0.010)
Population density	0.2604*** (0.000)	0.2562*** (0.000)	0.2737*** (0.000)	0.2990*** (0.000)	0.2758*** (0.000)	0.3047*** (0.000)	0.2233*** (0.001)
Age structure	27.601^a^ (0.310)	67.547^b^ (0.384)	25.849^c^ (0.179)	38.935** (0.038)	26.768 (0.157)	32.977* (0.077)	38.726** (0.047)
ShareW	224.9*** (0.000)	225.6*** (0.000)	219.2*** (0.000)		233.4 *** (0.000)	240.7*** (0.000)	226.9*** (0.001)
Industry	40.260*** (0.000)	40.899*** (0.000)	39.097*** (0.000)	40.039*** (0.000)	37.233*** (0.000)		
Services (share)						− 27.849*** (0.000)	
Tourism							− 5.7555 (0.812)
Poverty	10.781 (0.169)	11.208 (0.158)	10.984 (0.161)	15.498 * (0.052)		-0.028 (0.997)	5.929 (0.499)
GDP PPPpc					0.00176 (0.729)		
Education	10.022* (0.081)	10.215* (0.083)	10.733* (0.063)	9.135 (0.112)	6.680 (0.216)	10.383 * (0.067)	14.213 ** (0.021)
Constant	− 13,038 *** (0.000)	− 12,990 *** (0.000)	− 13,638 *** (0.000)	− 2233*** (0.000)	− 13,290*** (0.000)	− 11,214*** (0.001)	− 13,164*** (0.000)
ρ	0.3602*** (0.000)	0.3566*** (0.000)	0.3578*** (0.000)	0.4007*** (0.000)	0.3821*** (0.000)	0.3815*** (0.000)	0.4557*** (0.000)
λ	0.5237*** (0.003)	0.5338*** (0.002)	0.5255*** (0.003)	0.4928*** (0.003)	0.5508*** (0.001)	0.5035*** (0.004)	0.6045*** (0.000)
Pseudo R2	0.5014	0.5012	0.5024	0.4440	0.4780	0.4884	0.3293
Efficiency impact	1273.7** (0.029)	1180.7** (0.038)	1282.1** (0.026)	1404.0** (0.025)	1330.4** (0.027)	1353.6** (0.025)	1761.2** (0.010)

Each column presents the coefficient estimates (and respective p-values in parenthesis) for a given SARAR model

^a^Age structure indicator: percentage of the population aged 75 years or older

^b^Age structure indicator: percentage of the population aged 85 years or older

^c^Age structure indicator: median age of the population

***significant at the 1% level; **significant at the 5% level; *significant at the 10% level

## Alternative estimations: inverse distance spatial weights matrix (IDM); without Liechtenstein (LI); and estimated excess mortality rate (EM) as dependent variable

**Table:**

Variable	Model 13 (SAR with IDM)	Model 14 (SARAR with IDM)	Model 15 (SAR without LI)	Model 16 (SARAR without LI)	Model 17 (SAR with EM)	Model 18 (SARAR with EM)	Model 19 (OLS estimation)
Efficiency	1319.0*** (0.003)	1210.9*** (0.005)	1322.5** (0.001)	990.9** (0.021)	2145.6*** (0.000)	1411.2** (0.018)	1352.8*** (0.004)
Population density	0.1636** (0.047)	0.1315* (0.092)	0.3022*** (0.000)	0.2773*** (0.000)	0.2821*** (0.003)	0.2544*** (0.007)	0.2758*** (0.001)
Age structure	19.619 (0.326)	24.139 (0.225)	25.087 (0.161)	27.136 (0.142)	-13.226 (0.603)	-3.779 (0.885)	30.231 (0.147)
ShareW	262.27*** (0.000)	244.7*** (0.000)	212.4*** (0.000)	218.9*** (0.000)	436.5*** (0.000)	415.3*** (0.000)	262.6*** (0.000)
Industry	63.011*** (0.000)	58.022*** (0.000)	44.392*** (0.000)	39.320*** (0.000)	63.514*** (0.000)	61.054*** (0.000)	70.261 *** (0.000)
Poverty	19.170** (0.020)	20.812** (0.012)	13.654* (0.062)	11.432 (0.145)	29.042*** (0.005)	21.858** (0.048)	13.080 (0.124)
Education	6.148 (0.292)	6.740 (0.260)	8.850* (0.082)	10.051* (0.072)	22.637 *** (0.002)	20.135 ** (0.013)	11.449 * (0.053)
Constant	− 15,808*** (0.000)	− 14,992*** (0.000)	− 13,051*** (0.000)	− 13,079 *** (0.000)	− 25,733*** (0.000)	− 23,839*** (0.000)	− 16,012*** (0.000)
ρ	0.4711*** (0.002)	0.5922*** (0.001)	0.3878*** (0.000)	0.3727*** (0.000)	0.4550*** (0.000)	0.4352*** (0.000)	
λ		0.6977** (0.033)		0.419*** (0.005)		0.5374*** (0.001)	
Pseudo R2	0.4746	0.4251	0.5303	0.5105	0.6151	0.5792	0.4705
Efficiency impact	1915.2*** (0.006)	2016.3** (0.017)	1872.2** (0.001)	1378.3** (0.021)	3293.1** (0.000)	2111.5** (0.017)	1352.8*** (0.004)

Each column presents the coefficient estimates (and respective p-values in parenthesis) for alternative SAR or SARAR models: IDM – models estimated using an inverse distance spatial weights matrix (instead of a contiguity matrix); LI – models estimated without Liechtenstein; EM – models that use a constructed estimate of excess mortality as dependent variable (instead of C19deaths). The final line presents the estimate for the total impact of efficiency on the dependent variable ***significant at the 1% level; **significant at the 5% level; *significant at the 10% level

## Footnotes

1 Infant mortality was not used because it refers to the health of a small population segment (newly born), and a population segment that was barely affected by COVID-19 deaths. Nevertheless, robustness checks performed (see Section  “Results ”) showed that the inclusion of infant mortality in the analysis would not change the results significantly.

2 The NUTS classification (Nomenclature of territorial units for statistics) is a hierarchical system for dividing up the economic territory of the EU and associated countries, set up by Eurostat. See https://ec.europa.eu/eurostat/web/nuts/background for more information.

3 Excess mortality would likely have been a better indicator of health system resilience than COVID-19 death rates, but comparable data at a regional level were not available. For this robustness check, an “estimated excess mortality rate” was calculated using the ratio between excess mortality and reported COVID19 deaths for each country in 2020/21, which was then applied to the regional COVID19 deaths data. I.e., excess mortality for each region was estimated assuming that the discrepancy between excess mortality and COVID19 deaths was specific to each country and did not differ across regions of the same country.
